# Supplementary material for: Venomix: a simple bioinformatic pipeline for identifying and characterizing toxin gene candidates from transcriptomic data
Source: PeerJ. 2018 Jul 31;6:e5361. doi: 10.7717/peerj.5361 (PMC6074769; doi:10.7717/peerj.5361)
Supplement: Supplemental Information 3 [file peerj-06-5361-s003.gz › FinalOutput_E-6/Thrombin-like_enzyme_cerastobin_3/finaltree.pdf]

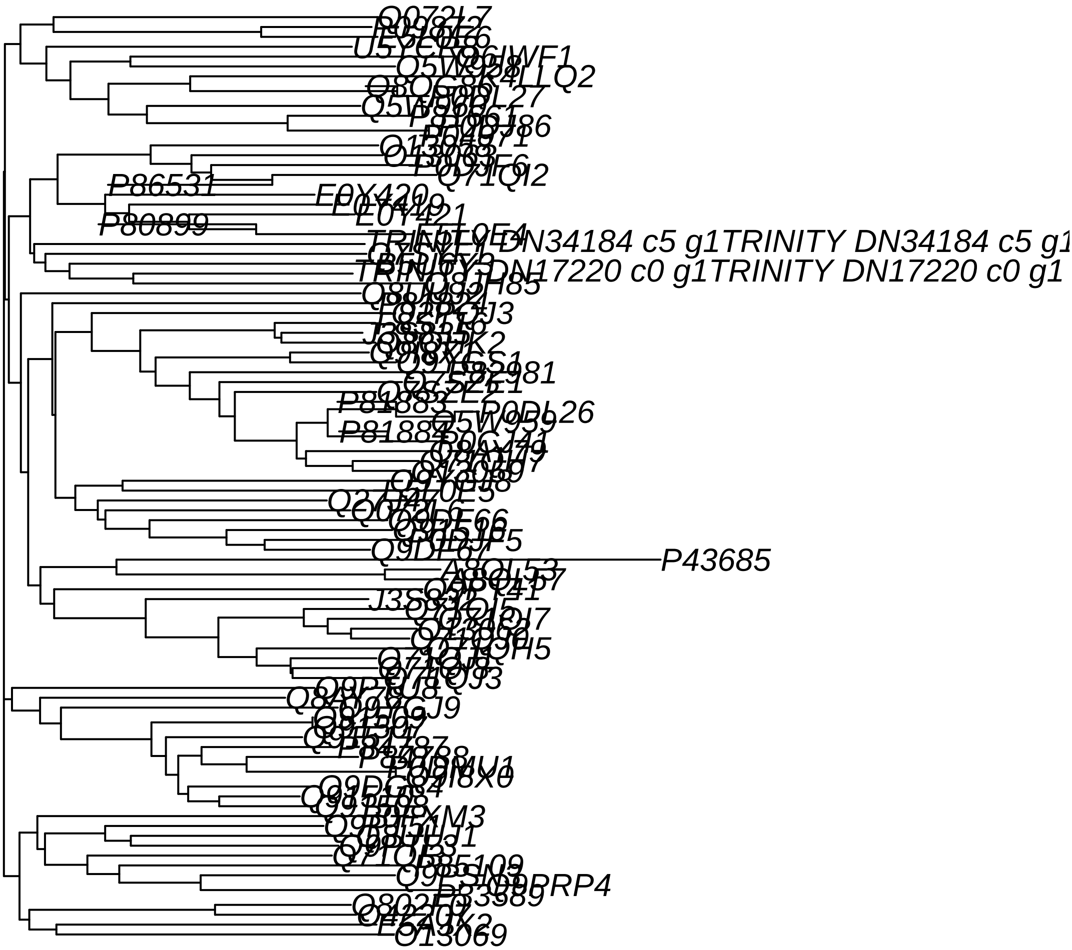

Q072L7
P86531
E0Y420
P80899
TRINITY DN34184 c5 g1TRINITY DN34184 c5 g1
TRINITY DN17220 c0 g1TRINITY DN17220 c0 g1
P81883
P81884
P43685
J3S832
Q71QI2
Q9PRP4
O13069
